# How are high burden countries implementing policies and tools for latent tuberculosis infection? A survey of current practices and barriers

**DOI:** 10.1002/hsr2.158

**Published:** 2020-05-03

**Authors:** Lena Faust, Morten Ruhwald, Samuel Schumacher, Madhukar Pai

**Affiliations:** ^1^ McGill International TB Centre Montreal General Hospital Montreal Quebec Canada; ^2^ Department of Epidemiology, Biostatistics and Occupational Health McGill University Montreal Qubec Canada; ^3^ Foundation for Innovative New Diagnostics (FIND) Geneva Switzerland

**Keywords:** latent tuberculosis, purified protein derivative, rifapentine, screening

## Abstract

**Background and aims:**

Despite the World Health Organization (WHO)'s updated guidelines on tuberculosis (TB) preventive treatment, the scale‐up of TB preventive therapy remains low in many high‐burden countries (HBCs). We conducted a survey to better understand the current status of policy implementation and barriers for scale‐up.

**Methods:**

Survey questions pertained to HBCs' current latent TB infection (LTBI) screening and treatment strategies, and the availability of LTBI tests and newer treatments (eg, isoniazid/rifapentine [3HP]). The 19‐question survey was piloted and sent out via email in June 2019 as a protected Microsoft Word document to contacts [National TB Program (NTP) staff, researchers, and health officials] in the 30 TB HBCs. Responses were accepted until February 2020.

**Results:**

Thirty‐seven completed surveys from 24 HBCs were received. Respondents from five countries (Brazil, Lesotho, Mozambique, Russia, Zambia) reported having LTBI guidelines that are fully implemented. Among respondents who indicated their country currently has no LTBI guideline implementation (Angola, China, DRC, India, Indonesia, Kenya, Myanmar), the most often cited barrier to implementation was the prioritization of active TB over LTBI management (n = 5, Angola, China, DRC, India, Kenya). Of the 16 countries in which respondents reported using purified protein derivative (PPD), 9 reported having experienced a PPD shortage within the past year (from time of survey). Respondents from six countries reported currently using Interferon‐gamma Release Assays (IGRAs) in their NTP, and 13 cited high cost as a barrier to IGRA use. Lastly, rifapentine was stated not be available in 8 HBCs.

**Conclusion:**

This survey indicates limited implementation of WHO LTBI guidelines in HBCs and provides some insight into barriers to implementation, including shortage of products (eg, PPD), high costs (eg, IGRAs), and lack of regulatory approval of newer treatments (eg, rifapentine). Thus, we should work towards price reductions for LTBI tests and treatments, and the development of tests that can be more easily implemented at peripheral healthcare levels.

## 1 INTRODUCTION

Tuberculosis (TB) continues to be a major global health concern, having resulted in an estimated 1.5 million deaths globally in 2018 ‐ more than any other infectious disease.^1^ Based on recent re‐estimations, approximately 23% of the global population has latent TB infection (LTBI),^2^ meaning that they have an immune response to *Mycobacterium tuberculosis* (*Mtb*), but only a small proportion will progress to active TB.^3^ Given the limitations of current evidence regarding optimal LTBI management, national policies regarding testing and treatment remain varied and inconsistently implemented. In particular, a review of 98 countries (including both high and low burden countries) found that algorithms for LTBI testing were inconsistently implemented, as were procedures for excluding active TB before initiating preventive treatment.^4^

While active TB continues to be a high priority for high‐burden countries (HBCs), these countries are starting to roll out TB preventive therapy. To this end, in 2020, the World Health Organization (WHO) issued updated guidelines on TB preventive treatment (module 1 of the 2020 WHO consolidated guidelines on TB), which include updated recommendations for HBCs.^5^ The guidelines comprise recommendations for targeted testing and treatment of people living with HIV (PLHIV), adults and children under 5 years of age who are household contacts of pulmonary TB cases, and HIV‐negative risk groups such as patients with silicosis, those on dialysis, or those receiving organ or haematological transplants.^5^ Importantly, as these groups are at increased risk of progression to active TB disease, the updated guidelines recommend targeting these groups for LTBI screening and treatment in all settings, independent of TB prevalence.^5^ Moreover, the 2020 guidelines state that systematic LTBI testing and treatment can also be considered in other risk groups, such as healthcare workers, prisoners, or the homeless, regardless of TB burden (as opposed to primarily in low‐burden settings as indicated in the 2018 LTBI management guidelines).^5^

The global TB community has ambitious targets for preventive therapy. The first‐ever United Nations General Assembly high‐level meeting on TB endorsed an ambitious political declaration, which commits to offering preventive therapy to at least 30 million people, including 4 million children under 5 years of age, 20 million other household contacts of people affected by TB, and 6 million PLHIV, by 2022.^6^

However, there are a myriad of challenges associated with both diagnosing and treating LTBI,^7^, ^8^ particularly in the HBC context, including the logistical barriers associated with requiring patients to return for Tuberculin Skin Test (TST) results to be read,^9^ and widespread Bacille Calmette‐Guérin (BCG) vaccination in HBC countries leading to high numbers of false positive TST results.^10^ Further challenges include a lack of new funding for LTBI programs, infrastructure requirements,^5^ shortages of products such as purified protein derivative (PPD),^11^ the unavailability of newer LTBI drugs (eg, rifapentine) in some countries,^12^, ^13^ and the unavailability of child‐friendly LTBI treatment formulations.^14^

In light of the abovementioned persisting challenges for LTBI screening and treatment in HBCs, it is of interest to investigate the extent to which HBCs are currently planning or able to implement the WHO's updated guidelines on LTBI management, and which barriers they face. This study, therefore, presents the results of a survey of HBC National TB Programs (NTPs), aiming to identify challenges experienced in HBCs with regards to the implementation of LTBI policies and tools.

## 2 METHODS

A survey was developed through the collaboration of TB experts from the Foundation For Innovative New Diagnostics (FIND) and the McGill International TB Centre, with the aim of better understanding plans and challenges regarding the introduction of new LTBI tests and treatment regimens in HBCs (with HBCs defined according to the WHO's high TB burden country list).^15^ As the survey collected only country‐level information and no individual data on human subjects, ethical approval was not required.

No specific sample size for the survey was targeted, although we aimed for at least one response from each of the 30 HBCs. Contact information for potential participants was compiled from prior TB‐related conferences or seminars (such as the McGill Summer Institute in Infectious Diseases and Global Health, held annually in Montreal, Canada, and the 2019 Meeting of the Working Group on Public‐Private Mix [PPM] for TB Care and Prevention, held in Jakarta, Indonesia).

Survey questions included both open‐ended and predefined response options, and where predefined options were given, a “specify other” field was always provided to allow any additional responses not captured in the given options. The questions were prepared and piloted among four respondents in May 2019, and pertained to the HBC's current LTBI screening and treatment strategy; the availability of Interferon‐gamma Release Assays (IGRAs), PPD, and various treatment regimens; and budget plans for future rollout of tests or treatments. The pilot‐phase respondents included health ministry officials and NTP staff, who were asked to provide their input on any unclear questions. As the questions were generally well understood, the survey was sent out in June 2019 (via email, as a protected Microsoft Word document) to HBC contacts, including NTP staff, researchers, and health officials. Contacts were informed of the purpose of the survey, and that the results would be summarized by country, with all participant names remaining anonymous and being treated as confidential. Up to four waves of reminders were sent to contacts that had not yet responded to the survey, with the last wave of reminders sent in November 2019. Responses were accepted until February 2020. Pilot‐phase responses were also included in the final analysis.

Survey data were extracted using a data extraction form created in Microsoft Excel (version 16.34). Data are presented descriptively by country. In the case of countries with multiple responses, all responses are taken into account in the presentation of data for that country (eg, if a respondent indicated financial barriers to LTBI guideline implementation and a second respondent from the same country indicated lack of staff as a barrier, then both are listed as barriers to LTBI guideline implementation for that country). In the case of directly conflicting responses between two respondents from the same country (eg, having vs not having a policy for 3HP implementation), the conflicting responses are indicated (eg, Table 7: conflicting responses regarding 3HP implementation indicated with an asterisk). For countries where respondents indicated having national LTBI guidelines in place, the existence of such guidelines was verified, where possible, through published papers or governmental policy documents.

## 3 RESULTS

### 3.1 Responses

The survey was sent to 128 contacts in the 30 HBCs, and 37 responses were received, from 24 different HBCs (Ethiopia (n = 7 respondents), Nigeria (n = 4), Pakistan (n = 3), Philippines (n = 2), India (n = 2), and Angola, Bangladesh, Brazil, Cambodia, China, the Democratic Republic of the Congo (DRC), Indonesia, Kenya, Lesotho, Liberia, Mozambique, Myanmar, Russia, South Africa, Tanzania, Thailand, Vietnam, Zambia, Zimbabwe (n = 1 respondent per country). Respondents included NTP managers or staff members (n = 22), staff of NGOs partnered with an NTP (n = 5), TB researchers or research center officials (n = 4), national TB reference laboratory staff (n = 3), health ministry officials (n = 1), and physicians (n = 2). Number of individuals contacted per country, responses received per country, and respondent affiliations by country are shown in Table 1.

**TABLE 1:** Number (N) of individuals contacted, number of respondents, and affiliation of respondents by country, for the 30 TB HBCs

WHO region	Country	N contacted	N responded	Affiliation of respondent(s) *						
AFR	Angola	2	1							
	Central African Republic	1	0							
	Congo	2	0							
	Democratic Republic of Congo	2	1							
	Ethiopia	15	7							
	Kenya	3	1							
	Lesotho	2	1							
	Liberia	1	1							
	Mozambique	3	1							
	Namibia	2	0							
	Nigeria	10	4							
	Sierra Leone	1	0							
	South Africa	10	1							
	Tanzania	6	1							
	Zambia	4	1							
	Zimbabwe	1	1							
AMR	Brazil	1	1							
EMR	Pakistan	9	3							
EUR	Russian Federation	3	1							
SEAR	Bangladesh	9	1							
	Democratic Republic of Korea	1	0							
	India	11	2							
	Indonesia	2	1							
	Myanmar	7	1							
	Thailand	3	1							
WPR	Cambodia	6	1							
	China	4	1							
	Papua New Guinea	2	0							
	Philippines	2	2							
	Vietnam	3	1							
	Total	128	37								

Abbreviations: AFR, African Region; AMR, American Region; EMR, Eastern Mediterranean Region; EUR, European Region; HBCs, high‐burden countries; SEAR, South‐East Asian Region; WPR, Western Pacific Region.

a One square per respondent.

**Table:** 

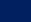 National TB Program manager or staff.
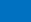 Non‐Governmental Organization (NGO) staff (partnered with NTP).
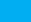 TB researcher or research center official.
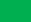 National TB reference laboratory staff.
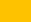 Physician.
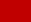 Health ministry official.
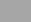 No respondents.

### 3.2 LTBI guideline implementation

Respondents of only five countries reported having national LTBI guidelines that are fully implemented (ie, all recommendations in the guidelines are carried out; Brazil, Lesotho, Mozambique, Russia, and Zambia). Respondents from seven countries indicated that their NTP does not have national LTBI guidelines (Angola, China, DRC, India, Indonesia, Kenya, Myanmar), and respondents from the remaining 12 countries reported that LTBI guidelines exist in their NTP, but that these are not fully implemented. Among the 17 countries for which respondents reported that national LTBI guidelines exist (either fully or partially implemented), these guidelines were available in the literature (scientific papers or governmental policy documents) for 14 countries (Ethiopia,^14^, ^16^ Lesotho,^17^ Mozambique,^18^ Nigeria,^19^ South Africa,^20^ Tanzania,^21^ Zambia,^22^ Zimbabwe,^23^ Thailand,^4^ Cambodia,^4^ Philippines,^24^ Vietnam,^4^ Brazil,^25^ and Pakistan^4^). LTBI management guidelines for all of these countries are outlined as part of their overall national TB guidelines, except in the case of Brazil, where LTBI guidelines are provided in a separate policy document specific to LTBI.^25^ Documents outlining national policies specific to the management of LTBI were not found for Liberia,^4^ Bangladesh,^26^ or Russia.

Among respondents who stated their countries lack LTBI guidelines, the most often cited barrier to guideline implementation was the prioritization of active TB over LTBI management (n = 5, Angola, China, DRC, India, Kenya). Other barriers included financial barriers to program implementation (n = 3, Angola, DRC, Kenya), lack of program staff (DRC), and guideline development still being in progress (n = 4, India, Indonesia, Kenya, Myanmar).

Among countries with respondents reporting existing LTBI guidelines that are, however, not yet fully implemented, financial barriers to program implementation were most commonly cited (n = 7, Ethiopia, Liberia, Nigeria, Pakistan, South Africa, Tanzania, Zimbabwe), followed by the prioritization of active TB (n = 6, Bangladesh, Ethiopia, Nigeria, Pakistan, South Africa, Tanzania), and a lack of program staff (n = 4, Cambodia, Liberia, Pakistan, Zimbabwe) or insufficient training of staff (Bangladesh). Other barriers mentioned were policy development still being recent or ongoing (n = 2, Vietnam, Philippines), a lack of public understanding and willingness (for screening; Thailand), a lack of program coordination and inadequate knowledge to write guidelines (Ethiopia), the heavy workload of healthcare workers and the fact that not all necessary supplies are available regularly (Philippines), and concerns regarding contributing to the development of resistance to isoniazid (INH; Pakistan).

### 3.3 LTBI screening and treatment practices

Considering the risk groups in which LTBI screening is recommended in high burden settings by the updated 2018 WHO guidelines,^4^ only the respondent from China reported not targeting any of these groups for LTBI screening or treatment. Overall, respondents from 23 countries reported that people living with HIV are targeted for LTBI treatment in their NTP (all except China), 22 reported targeting under 5‐year‐old household contacts of bacteriologically confirmed pulmonary TB cases (all except China and Angola), and 14 reported targeting those aged 5 or above who are household contacts of a bacteriologically confirmed pulmonary TB case (all except Angola, Bangladesh, Cambodia, China, India, Indonesia, Kenya, Myanmar, the Philippines, and Tanzania). Tables 2 and 3 display screening tools and treatment regimens used by NTPs in these and other risk groups, as reported by the respondents.

**TABLE 2:** LTBI screening tools and treatment regimens used in people living with HIV and in household contacts of bacteriologically confirmed pulmonary TB cases in 24 high TB burden countries according to survey respondents

Country	Screening tools used					Treatment administered				
	IGRA	TST	CXR	Other	Clinical screening only, to rule out active TB (a)	INH	RIF	RIF + INH	3HP	Other
Risk group: People living with HIV*										
Angola										
Bangladesh										
Brazil										
Cambodia										
China										
DRC										
Ethiopia										RH 6 m
India										
Indonesia										
Kenya					(b)					
Lesotho										
Liberia										
Mozambique										
Myanmar										
Nigeria										
Pakistan				GeneXpert						
Philippines										
Russia				DiaskinTest						
South Africa										
Tanzania										
Thailand										
Vietnam				GeneXpert					(c)	
Zambia										
Zimbabwe										
Risk group: Those <5 years of age who are contacts of a bacteriologically confirmed pulmonary TB case*										
Angola										
Bangladesh										
Brazil										
Cambodia										
China										
DRC										
Ethiopia										RH 6 m
India										
Indonesia										
Kenya					(d)					
Lesotho										
Liberia										
Mozambique										
Myanmar										
Nigeria										
Pakistan										
Philippines										
Russia				DiaskinTest						RZ 3‐6 m
South Africa										
Tanzania										
Thailand										
Vietnam									(c)	
Zambia										
Zimbabwe										
Risk group: Those ≥5 years of age who are contacts of a bacteriologically confirmed pulmonary TB case*										
Angola										
Bangladesh										
Brazil										
Cambodia										
China										
DRC										
Ethiopia										RH 6 m
India										
Indonesia										
Kenya										
Lesotho										
Liberia										
Mozambique										
Myanmar										
Nigeria										
Pakistan				GeneXpert					(e)	
Philippines										
Russia				DiaskinTest						RZ 3‐6 m
South Africa										
Tanzania										
Thailand										
Vietnam									(c)	
Zambia										
Zimbabwe										

Abbreviations: 3HP, rifapentine and isoniazid weekly for 3 months; CXR, chest X‐Ray; IGRA, interferon gamma release assays; INH, isoniazid daily for 6‐9 months; LTBI, latent TB infection; RH 6 m, rifampicin and isoniazid for 6 months; RIF, rifampicin daily for 3‐4 months; RIF + INH, rifampicin and isoniazid daily for 3‐4 months; RZ 3‐6 m, rifampicin and pyrazinamide for 3‐6 months; TST, tuberculin skin test.

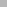
 Risk group is not screened in NTP.

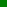
 Screening tool/treatment regimen is used in this risk group in NTP.

(a) May include the use of nontechnological “tools,” such as symptom scores or questionnaires; (b) CXR where available, but clinical screening only is the more common scenario; (c) Will gradually expand 3HP and 3RH implementation; (d) TST and CXR where available, especially in private facilities, but clinical screening only is the more common scenario; (e) Rifapentine in trial phase in selected areas.

a These represent risk groups in which LTBI screening is recommended in high burden settings as per the 2018 WHO guidelines (in effect at the time of the survey), and remain unchanged in the updated 2020 guidelines, which now supersede those from 2018.^5^

**TABLE 3:** LTBI screening tools and treatment regimens used in other risk groups targeted for LTBI screening in 24 high TB burden countries according to survey respondents

Country	Other risk groups targeted for LTBI screening												LTBI screening tools used					LTBI treatment regimens used				
	Healthcare workers ^a^	Transplant recipients ^b^	On dialysis /anti‐TNF ^b^	Silicosis patients ^b^	Diabetes patients	Migrants	Prisoners ^a^	Drug users ^a^	Alcohol users	Tobacco users	Underweight individuals	Other	IGRA	TST	CXR	Other	Clinical screening only (a)	INH	RIF	RIF + INH	3HP	Other
Angola																						
Bangladesh																						
Brazil																						
Cambodia																						
China																						
DRC																						
Ethiopia												Mining staff, university attendees										
India																						
Indonesia												Unspecified										
Kenya																						
Lesotho																						
Liberia																						
Mozambique																						
Myanmar																						
Nigeria																GeneXpert						
Pakistan																GeneXpert						
Philippines																						
Russia																Diaskin‐Test						RZ 3‐6 m
South Africa												Unspecified										
Tanzania												Contacts of MDR‐TB cases				GeneXpert						(b)
Thailand																						
Vietnam												Employees of congregate settings				GeneXpert					(c)	
Zambia																						
Zimbabwe																						

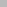
 Risk group is not screened in NTP.

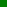
 Risk group is screened/screening tool/treatment regimen is used in this risk group in NTP.

(a) May include the use of nontechnological “tools,” such as symptom scores or questionnaires; (b) Levofloxacin or Moxiflocacin for 6 to 9 months for MDR contacts; (c) Will gradually expand 3HP and 3RH implementation.

Abbreviations: 3HP, rifapentine and isoniazid weekly for 3 months; CXR, chest X‐ray; IGRA, interferon gamma release assays; INH, isoniazid daily for 6 to 9 months; LTBI, latent TB infection; MDR‐TB, multidrug‐resistant tuberculosis; NTP, national TB program; RH 6 m, rifampicin and isoniazid for 6 months; RIF, rifampicin daily for 3 to 4 months; RIF + INH, rifampicin and isoniazid daily for 3 to 4 months; RZ 3‐6 m, rifampicin and pyrazinamide for 3 to 6 months; TST, tuberculin skin test.

a These represent risk groups in which LTBI screening can be considered in high burden settings, as per the 2020 WHO guidelines (not yet in effect at the time of the survey).^5^

b These represent risk groups in which LTBI screening is recommended in high burden settings as per the 2018 WHO guidelines (in effect at the time of the survey) and remain unchanged in the 2020 updated guidelines, which now supersede those from 2018.^5^

### 3.4 PPD use, availability, and costs

Respondents from only three countries (China, Indonesia, and Russia) reported having a local manufacturer of PPD within their country (BioFarma in Indonesia, the Research Institute of Vaccines and Serums (St. Petersburg) and Pharmstandart Pharmaceutical (Moscow) in Russia, and multiple manufacturers in China, including Beijing Sanroad Biological Products and Beijing Wantai Biological Pharmacy Enterprise). Of the 24 HBCs responding to the survey, respondents from 16 countries reported having at least one type of PPD approved for use in their NTP, while three (Cambodia, Myanmar, and Tanzania) stated not knowing whether PPD is approved for use in their country. Respondents from eight countries reported not using PPD in their NTP (Angola, Cambodia, DRC, Lesotho, Liberia, Tanzania, Russia, and Zambia). Of these, four (Angola, DRC, Lesotho, and Liberia) stated that no type of PPD is currently approved for use in their country.

Among the remaining 16 countries whose respondents reported using PPD, the most commonly used types were PPD RT23, produced by AJ Vaccines, Denmark (previously Statens Serum Institut; n = 7, Brazil, India, Pakistan, Philippines, Thailand, Vietnam, Zimbabwe), and PPD‐S2: Tubersol, by Sanofi Pasteur (n = 3, Kenya, South Africa, Zimbabwe). Further PPD types approved in NTPs, as reported by the respondents, are shown in Table 4. Respondents from nine countries reported having experienced a PPD shortage within the past year, while respondents from four countries (Bangladesh, Ethiopia, Myanmar, and Nigeria) stated not knowing whether a shortage was experienced. Of the three countries whose respondents did not report a PPD shortage (China, Indonesia, and Mozambique), two (China and Indonesia) reported using locally‐manufactured PPD. PPD shortage status by country, as reported by the respondents, is shown in Table 4.

**TABLE 4:** PPD availability in 24 high‐TB‐burden countries according to survey respondents

Country	Within‐country manufacturer	Type of PPD approved/registered in NTP (and manufacturer)						PPD shortage within the past year*
		PPD RT23 (AJ Vaccines)	PPD‐S2: Tubersol (Sanofi Pasteur)	PPD‐s (Nippon BCG Seizo)	PPD (SPAN diagnostics/Arkray Healthcare, India)	Tuberculin PPD BNCIPD (Bulgaria)	Other	
Angola								
Bangladesh								
Brazil								
Cambodia							(a)	
China							PPD (Beijing Sanroad Biological Products, China) (b)	
DRC								
Ethiopia								
India								
Indonesia							PPD RT23 (Biofarma)	
Kenya		(c)						
Lesotho								
Liberia								
Mozambique							PPD Aplisol (Par Pharmaceuticals, EU)	
Myanmar							(a)	
Nigeria								
Pakistan								
Philippines								
Russia	(d)						PPD‐L(Linnikova)‐2 (b)	
South Africa								
Tanzania							(a)	
Thailand								
Vietnam								
Zambia							PPD RT23 (Evans PPD; Celltech Pharma, Spain)	
Zimbabwe								

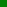
 Type of PPD approved/registered in country. 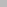 PPD not used in NTP. PPD shortage (NTP has experienced a PPD shortage within the past year): 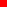 Yes 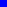 No 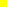 Unsure.

(a) Unsure if any type of PPD is registered in the country; (b) ESAT6‐CFP10‐based skin tests are used in China (EC, Anhui Zhifei Longkom Biopharmaceutical, China) and Russia (Diaskintest, Generium Pharmaceutical, Russia); (c) PPD RT23 is approved, but unsure which manufacturer; (d) Within‐country manufacturer, although PPD not used in NTP.

Abbreviations: BNCIPD, Bulbio National Centre for Infectious and Parasitic Diseases, Bulgaria; NTP, national TB program; PPD, purified protein derivative.

a From time of survey.

Of the nine countries whose representatives reported experiencing a PPD shortage, most (n = 5, Brazil, India, Pakistan, Zimbabwe, and Thailand) cited delayed or insufficient supply from the manufacturer as the main reason for the shortage. Additional reasons mentioned included not having a supplier (Philippines), the supplier ceasing importation (Vietnam), and the supplier's authorization from the manufacturer not being renewed (Pakistan, manufacturer: Sanofi Pasteur). In the case of Kenya, it was also mentioned that staffs are sometimes not aware when PPD does become available, leading to further delays in implementation.

**FIGURE 1:**
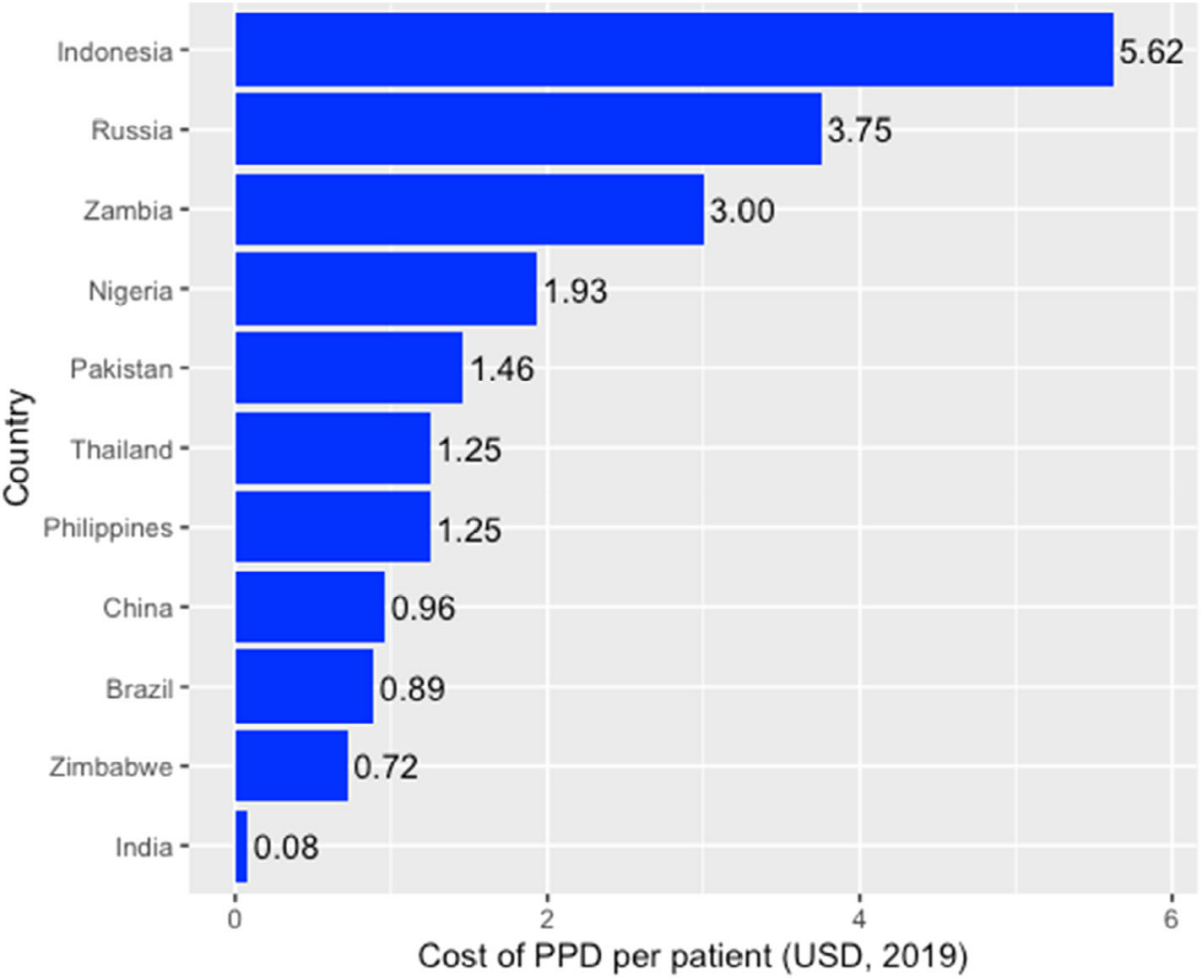
Costs of purified protein derivative (PPD) per patient (USD) in high‐TB‐burden countries

Representatives from 11 countries were able to provide cost estimates for PPD in their NTPs. Costs per patient are shown in USD in Figure 1.

### 3.5 IGRA policies and implementation

Respondents from 6 of the 24 HBCs reported that IGRAs are currently used in their NTP (Cambodia, China, Nigeria, Russia, Tanzania, and Thailand). Respondents from 10 countries specified having a national policy on the use of IGRAs for LTBI screening (Brazil, Cambodia, Ethiopia, Nigeria, Pakistan, Russia, Tanzania, Thailand, Zambia, and Zimbabwe), however, 5 of these 10 reported that IGRAs are not currently used in their NTP (Brazil, Ethiopia, Pakistan, Zambia, Zimbabwe). Moreover, respondents report that NTP policies in Ethiopia and Pakistan currently do not recommend the use of IGRAs in the local epidemiological context, and Brazil's NTP is awaiting national approval for IGRA use.

Considering the six NTPs in which respondents reported currently using IGRAs, the IGRAs used are QuantiFERON‐TB Gold Plus (QFT‐Plus; Qiagen, Germany) in Nigeria and Thailand; QFT Gold In Tube (QFT‐GIT; Qiagen) in China, Nigeria, and Russia; TB‐IGRA (Beijing Wantai, China) in Tanzania and China; T‐SPOT.TB (Oxford Immunotec, UK) in China, Thailand, and Russia; and QB‐SPOT (Beijing Kinghawk) in China (which also reported using other (unspecified) domestically manufactured IGRAs). The specific IGRAs used in Cambodia were not specified.

Respondents from the majority of countries surveyed reported significant barriers to IGRA implementation in their NTPs. The most frequently reported barriers are financial, with respondents from most countries reporting the high cost of IGRAs (n = 13) and the lack of allocation of a budget to IGRA implementation (n = 11) as current barriers for their NTP. Among those indicating the lack of an allocated budget for IGRAs, respondents from only one country (Ethiopia) stated having plans for a budget. Other common barriers among NTPs included insufficient capacity for specimen transport (n = 9), lack of laboratory infrastructure for IGRA implementation (n = 10), domestic unavailability of IGRA kits (n = 6), and limited availability of laboratory personnel (n = 6). Barriers to IGRA implementation experienced in each NTP are shown in Table 5. Respondents from Russia and the DRC were the only ones not reporting any barriers to IGRA implementation.

**TABLE 5:** Barriers to implementation of IGRAs for LTBI screening in HBC NTPs according to survey respondents

	Country	Barriers to IGRA implementation							
		High cost	No budget allocated	Not clear if IGRAs better than PPD	IGRA kits not domestically available	Lack of laboratory infrastructure	Limited availability of laboratory personnel	Insufficient capacity for specimen transport	Other
Currently using IGRAs	Cambodia								
	China								
	Nigeria								
	Tanzania								
	Thailand								
	Russia								
Currently not using IGRAs	Brazil								Awaiting approval for use of IGRAs (by National Committee for New Technologies Incorporation)
	Angola								
	Bangladesh								
	DRC								
	Ethiopia								NTP policy does not recommend use of IGRAs
	India								Lack of country‐specific evidence for IGRA cut‐offs
	Indonesia								Lack of policy on IGRA use
	Kenya								Policy on IGRA use still in development
	Lesotho								
	Liberia								
	Mozambique								
	Myanmar								
	Pakistan								NTP policy does not recommend use of IGRAs
	Philippines								Requirement for blood draw
	South Africa								
	Vietnam								Complexity of technique and lack of feasibility to apply at peripheral levels
	Zambia								LTBI previously not prioritized
	Zimbabwe								

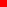
 Barrier experienced in NTP.

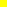
 Unsure if barrier is experienced in NTP.

Abbreviations: HBCs, high‐burden countries; IGRAs, interferon‐gamma release assays; LTBI, latent TB infection; NTP, national TB program; PPD, purified protein derivative.

Respondents from 12 countries provided estimates of how many individuals their NTP planned to screen for LTBI in 2019, via any test (see Table 6), however, only respondents from Thailand and Russia were able to provide estimates of how many are to be screened specifically with IGRAs. Cost estimates for IGRAs were also provided only from Thailand and Russia, reporting costs of 45 USD and 106 USD per patient, respectively.

**TABLE 6:** Number of individuals HBC NTPs plan to screen for LTBI (via any test, in 2019, unless otherwise specified), according to survey respondents

Country	Number of individuals NTP plans to screen for LTBI
Russia	24.1 million (3.6 million with IGRAs)
Pakistan	160 000 000
Nigeria	900 000 (by 2022)
Brazil	200 000
Philippines	169 139
Bangladesh	95 300
Thailand	90 000 (5000 with IGRAs)
Vietnam	30 941
Mozambique	29 000
Ethiopia	27 837
Indonesia	16 122*
Liberia	1836

Abbreviations: HBCs, high‐burden countries; IGRAs, interferon‐gamma release assays; LTBI, latent TB infection; NTP, national TB program; PPD, purified protein derivative.

a This represents the NTP's target of screening 15% of children under 5 with LTBI.

### 3.6 3HP implementation

Respondents from 13 countries reported having a policy on the implementation of the 3HP regimen (see Table 7). Respondents from most countries (n = 15) reported the high cost of the regimen as a barrier to implementation (including both countries with and without a current policy for 3HP use), and, as reported by the respondents, Rifapentine is not currently registered in eight countries. Other barriers to 3HP implementation in NTPs include concerns about drug‐drug interactions between Rifapentine and antiretrovirals (n = 7) and adverse effects of the regimen (n = 5). Again, respondents from the DRC and Russia did not report experiencing any barriers to implementation. Barriers reported in each NTP are shown in Table 7.

**TABLE 7:** Barriers to the implementation of the 3HP regimen in HBC NTPs according to survey respondents

	Country	Barriers to 3HP implementation							
		High cost	No budget allocated	If no budget is allocated, are there plans for a budget?	NTP does not treat latent infection	Rifapentine is not registered in the country	Concerns about adverse effects of the regimen	Concerns about DDI between rifapentine and ARVs	Other
NTP has a policy for 3HP implementation	Cambodia								
	Ethiopia*								3HP adoption is not planned
	Indonesia								
	Lesotho								
	Liberia			(a)					Lack of training among healthcare workers.
	Myanmar								
	Nigeria*								Availability of the regimen. Policy discussions regarding implementation are ongoing.
	Pakistan*								
	Russia								
	Thailand								
	Vietnam								
	Zambia								
	Zimbabwe								Lack of funding for the training of healthcare workers.
No current policy for 3HP implementation	Angola								
	Bangladesh								Lack of policy for implementation.
	Brazil								
	China								
	DRC								
	India								
	Kenya			(b)					
	Mozambique								
	Philippines								
	South Africa								
	Tanzania								Coordination issues with national AIDS program. In initial stages of pilot studies of implementation.

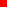
 Barrier experienced in NTP ARV Antiretrovirals.

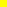
 Unsure if barrier is experienced in NTP DDI Drug‐drug interactions.

Abbreviations: HBCs, high‐burden countries; NTP, national TB program.

(a) Currently updating national strategic plan, 3HP will be budgeted; (b) 3HP is in the process of being procured, and will run as a pilot before nation‐wide roll out.

a Multiple respondents per country with differing responses regarding status of 3HP policy.

## 4 DISCUSSION

This survey represents a comprehensive overview of the status of barriers to the implementation of LTBI management guidelines in HBCs in 2019. It is first notable that while respondents from seven countries reported that their NTPs currently do not have LTBI management guidelines, respondents from only five countries reported having guidelines that are fully implemented, while 12 had guidelines that were not fully implemented. This suggests that a primary hurdle for the majority of countries may be simply getting targeted treatment of LTBI on the agenda. Indeed, although the main barrier to guideline implementation, as reported by the survey participants, among countries with reportedly no current guidelines was the prioritization of active TB, the major barriers among those with partially implemented guidelines were both cost and prioritization of active TB. This suggests that the prioritization of active TB is both an initial barrier for countries to establish guidelines in the first place, and a persisting barrier for countries with existing guidelines. The full implementation of these guidelines, once established, is then often hindered by financial challenges.

As respondents in most countries generally reported screening for LTBI in risk groups identified in previous WHO LTBI guidelines, such as in PLHIV, fewer stated that their country screened or treated in those groups identified in the 2018 guidelines, such as in individuals aged ≤5 years who are household contacts of bacteriologically confirmed pulmonary TB cases (note that this recommendation remains unchanged in the 2020 updated guidelines that now supersede those from 2018).^5^ This suggests that in the context of limited financial resources or supply of LTBI screening tools, HBCs may restrict their screening efforts to certain priority risk groups, and considerable additional resources and support may be required for HBCs to reach full guideline implementation in light of the expanded recommendations.

Furthermore, regarding the use and availability of PPD, respondents from nine countries reported having experienced a PPD shortage within the past year. In addition, the challenge of ensuring high PPD quality also underlines the need to invest further in the development of new LTBI tests that are non‐PPD‐based. It also indicates a need for further studies on the diagnostic performance of already available non‐PPD‐based skin tests, such as the Diaskintest and C‐Tb, to provide an evidence‐base for their potential as alternatives to PPD‐based tests.

Regarding the use of IGRAs as an alternative to the TST for LTBI testing, it is important to note that respondents from the majority of countries surveyed (13 out of 24) report financial barriers as a major hindrance to IGRA implementation. To address this, in December 2019, the Global Drug Facility included QFT‐Plus on its catalog, at a GDF‐negotiated price of $15.90 per test for HBCs.^27^ However, beyond just unit costs, respondents from many countries also reported the challenges of specimen transport and the lack of laboratory infrastructure as significant barriers to the use of IGRAs in their NTP. This requirement for laboratory facilities for IGRA implementation is also underlined in the 2020 WHO guidelines on TB preventive treatment as an ongoing operational barrier faced by countries with regards to LTBI management.^5^ This highlights the importance of continued efforts to develop new LTBI testing tools that do not require significant laboratory facilities or highly trained personnel, and that can be implemented at the peripheral healthcare level (other than the TST).

Given existing ESAT6/CFP10‐specific skin tests such as C‐Tb and the Diaskintest,^28^, ^29^ as well as the ongoing development of newer, simpler, point‐of‐care IGRAs,^30^ including the new QuantiFERON‐TB Access (QFT Access; Qiagen, Hilden, Germany), which can be implemented without the need for laboratory facilities,^31^ the TST may become less dominant in LTBI testing practices in low‐ and middle‐income countries (LMICs) in the future. Furthermore, partnerships such as that recently established between Statens Serum Institut (SSI, Denmark) and Serum Institute of India Pvt Ltd (SIIPL), allowing SIIPL to produce and distribute SSI's C‐Tb test,^32^ are promising steps toward improving the availability and accessibility of high‐quality LTBI tests. Given the mentioned shortcomings of the TST for LTBI testing, including the requirement for return visits by patients^9^ and its limited utility in settings where routine BCG vaccination continues,^7^, ^10^ the development of accessible alternatives to the TST is an important element of facilitating LTBI management.

With regard to LTBI treatment, representatives from 15 countries reported the high cost of the new 3HP regimen as a barrier to its implementation in their NTPs. In October 2019, Unitaid, the Global Fund, and Sanofi announced an almost 70% reduction in price for the 3HP regimen (from ~US$45 to US$15) in the public sectors of 100 LMICs with a high burden of TB and HIV/TB co‐infection.^33^ However, as called for in a civil society statement released at the 2019 Union World Conference on Lung Health in Hyderabad, India,^13^ it is imperative that the price of the regimen be further lowered.

Financial barriers aside, increased efforts must also be made to remove the administrative barriers to 3HP implementation that persist in many countries, including the fact that it was reported to remain unregistered for use in eight of the HBCs surveyed. Furthermore, patent applications for both pediatric and adult formulations of 3HP have been filed by Sanofi in 10 of the countries included in this survey (China, Indonesia, India, Philippines, Vietnam, Thailand, Nigeria, Brazil, the Russian Federation, and South Africa).^34^ Of these, the patent has been granted in the Russia Federation and in South Africa (valid until 2034), and has so far been rejected only in China, and withdrawn (by Sanofi) in Indonesia. In India and the Philippines, the filing of the application has been opposed, but a final decision has not yet been made. Patents applications in Vietnam, Thailand, Nigeria, and Brazil remain filed (pending a decision).^34^ Therefore, ongoing advocacy for patent opposition, particularly in high‐burden countries, may speed up the introduction of cheaper generics and reduce access barriers.

Lastly, given the variation in guideline implementation for LTBI management, efforts to simplify and standardize testing algorithms may facilitate introduction and adherence to these guidelines at national levels. In the longer term, this will also allow more comprehensive evaluation (and subsequent improvement) of current LTBI management policies.^4^

### 4.1 Limitations of the survey

No responses were received from 6 (20%) of the 30 HBCs, and of the 24 countries for which responses were received, 19 (79%) had only one respondent. One limitation of this survey is, therefore, the fact that 100% coverage of all 30 HBCs was not achieved, and furthermore, as there were different numbers of respondents across countries, overall results may be more comprehensive for countries with multiple respondents compared to those with only one respondent. In addition, given the nature of the study as a survey among NTP contacts, it must be emphasized that respondents' reports regarding LTBI management reflect only the respondents' current knowledge, and not necessarily the official situation in their respective countries. Moreover, reporting bias may have affected the accuracy of responses, as respondents may be inclined to report aspirational practices or plans rather than responses that most closely reflect current practice. Moreover, given that contact information for potential respondents was obtained through participant lists at prior TB‐related conferences, the results of the survey may not be representative of other TB experts in the country. Lastly, respondents held different positions and roles within their respective countries (eg, NTP manager vs TB researcher), so their scopes of expertise and available information may differ.

## 5 CONCLUSION

In conclusion, in light of the 2020 updated WHO guidelines on TB preventive treatment, which include new recommendations that apply to HBCs,^5^ our survey has identified some of the challenges that HBC NTPs face with regards to the full implementation of these guidelines. The findings suggest a need for price reductions for LTBI tests and treatments, the development of tests that can be easily implemented at peripheral healthcare levels, and overall, mechanisms to efficiently deliver to HBCs a comprehensive set of LTBI management tools, including both tests and treatment regimens.

## FUNDING INFORMATION

This work was supported by the Bill & Melinda Gates Foundation [OPP1061487] and FIND, 73 Geneva. The Bill & Melinda Gates Foundation or its employees did not play a role in study 74 design, data collection, data analysis, interpretation, writing of the manuscript, or submission 75 for publication. Two of the study authors are employed at FIND (MR & SS), and their 76 respective involvement in the study is described under “author contributions” above, and 77 conflicts of interests disclosed in the section below.

## AUTHOR CONTRIBUTIONS

Conceptualization: Madhukar Pai

Data Curation: Lena Faust

Formal Analysis: Lena Faust

Funding Acquisition: Morten Ruhwald, Samuel Schumacher, Madhukar Pai

Methodology: Madhukar Pai

Supervision: Madhukar Pai

Writing – Original Draft Preparation: Lena Faust

Writing – Review & Editing: Morten Ruhwald, Samuel Schumacher, Madhukar Pai

All authors have read and approved the final version of the manuscript. Lena Faust had full access to all of the data in this study and takes complete responsibility for the integrity of the data and the accuracy of the data analysis.

## CONFLICT OF INTEREST

L. F. has no competing interests to disclose. M. R. and S. S. are employed at FIND, a not‐for‐profit foundation evaluating diagnostics for TB and other diseases, with FIND's neutrality regarding these evaluations clearly established in product evaluation agreements. M. P. has previously served as a consultant for the Bill & Melinda Gates Foundation, and serves on FIND's Scientific Advisory Committee. These conflicts did not affect the study design, analysis, or interpretation.

## TRANSPARENCY STATEMENT

The lead author, Lena Faust, affirms that this manuscript is an honest, accurate, and transparent account of the study being reported; that no important aspects of the study have been omitted; and that any discrepancies from the study as planned have been explained.

## Data Availability

Lena Faust had full access to all of the data in this study and takes complete responsibility for the integrity of the data and the accuracy of the data analysis. The authors confirm that the data supporting the findings of this study are available within the article.
